# 

**Published:** 1864-02

**Authors:** 


Volume XXI. Number 2.

### THE CHICAGO MEDICAL JOURNAL.

DE LASKIE MILLER, M. D.,
EPHRAIM INGALS, M. D.,
Editors and Proprietors.

FEBRUARY, 1864.

CHICAGO:

J. C. W. BAILEY, BOOK AND JOB PRINTER, 130 CLARK STREET.

1864.

